# Competencies for first year residents – physicians’ views from medical schools with different undergraduate curricula

**DOI:** 10.1186/s12909-017-0998-9

**Published:** 2017-09-07

**Authors:** Sophie Fürstenberg, Kristina Schick, Jana Deppermann, Sarah Prediger, Pascal O. Berberat, Martina Kadmon, Sigrid Harendza

**Affiliations:** 1 Department of Internal Medicine, University Medical Center Hamburg-Eppendorf, Hamburg, Germany; 2 TUM Medical Education Center, School of Medicine, Technical University of Munich, Munich, Germany; 3 Department of Medical Education and Education Research, Carl von Ossietzky University of Oldenburg, Oldenburg, Germany; 4 Universitätsklinikum Hamburg-Eppendorf, III. Medizinische Klinik, Martinistr, 52 D-20246 Hamburg, Germany

**Keywords:** Competence, Curriculum, Internal medicine, Physician, Postgraduate medical education, Residency, Surgery, Undergraduate medical education

## Abstract

**Background:**

Frameworks like the CanMEDS model depicting professional roles and specific professional activities provide guidelines for postgraduate education. When medical graduates start their residency, they should possess certain competencies related to communication, management and professionalism while other competencies will be refined during postgraduate training. Our study aimed to evaluate the relevance of different competencies for a first year resident required for entrustment decision from the perspective of physicians from medical faculties with different undergraduate medical curricula.

**Methods:**

Nine hundred fifty-two surgeons and internists from three medical schools with different undergraduate medical curricula were invited to rank 25 competencies according to their relevance for first year residents. The rankings were compared between universities, specialties, physicians’ positions, and gender.

**Results:**

Two hundred two physicians participated, 76 from Hamburg University, 44 from Oldenburg University, and 82 from Technical University Munich. No significant differences were found regarding the top 10 competencies relevant for first year residents between the universities. ‘Responsibility’ was the competency with the highest rank overall. Internists ranked ‘Structure, work planning and priorities’ higher while surgeons ranked ‘Verbal communication with colleagues and supervisors’ higher. Consultants evaluated ‘Active listening to patients’ more important than department directors and residents. Female physicians ranked ‘Verbal communication with colleagues and supervisors’ and ‘Structure, work planning and priorities’ significantly higher while male physicians ranked ‘Scientifically and empirically grounded method of working’ significantly higher.

**Conclusions:**

Physicians from universities with different undergraduate curricula principally agreed on the competencies relevant for first year residents. Some differences between physicians from different positions, specialties, and gender were found. These differences should be taken into account when planning competence-based postgraduate education training programs.

## Background

Competencies play an increasing role in postgraduate medical education to define the tasks, which can be entrusted to residents at different stages and levels of their training [1, 2]. With the CanMEDS model developed by the Royal College of Physicians and Surgeons of Canada, a widely accepted framework based on competencies allocated to seven roles of a physician was established for postgraduate education [3]. Competency-based postgraduate trainings based on CanMEDS have evolved meanwhile in different countries and for different specialties, e.g. internal medicine and surgery [4, 5]. Canadian directors for postgraduate medical education programs believe though that the CanMEDs roles and their underlying competencies are difficult to teach and assess [6]. In this context, a feasibility study has been performed to develop entrustable professional activities as the basis for assessment of competence [7]. However, the integration of specific competencies (e.g. cultural competency) or competencies required for certain entrustable professional activities into residency training programs for surgery or internal medicine, which are accredited by the Accreditation Council for Graduate Medical Education (ACGME), is not trivial [8, 9] and requires an adaptation in educational culture by the physicians involved as supervisors.

To ease the transition from undergraduate to postgraduate medical training, medical schools are switching from traditional curricula to vertically integrated or competency-based undergraduate medical education [10, 11]. Students from medical schools with vertically integrated curricula needed less time and fewer applications to obtain a residency position and felt better prepared for work and postgraduate training than students who followed a traditional curriculum [12, 13]. If the CanMEDS framework is used to design a competency-based catalogue of learning objectives for undergraduate medical education, curriculum planners need to be aware that teachers’ perceptions of competencies can differ extensively [14]. A ranking study of competencies required for the first year of residency performed among physician educators in the Netherlands and Germany showed full agreement about the top 10 competencies [15]. However, the perspectives on competencies needed for the first year of residency might differ among physician educators according to the curricular structure of the undergraduate training they are used to or according to their specialty or position within a hospital. The aim of our study was to assess whether such differences could be identified between physicians of different specialization and from medical schools with different undergraduate medical curricula.

## Methods

To determine the perspective of teaching physicians on the importance of different competencies for entrustment decisions in first year residents, physicians from three German medical schools with different undergraduate medical curricula participated in this survey. The University Medical Center Hamburg-Eppendorf (UKE) has a vertically integrated undergraduate model curriculum, the Carl von Ossietzky University of Oldenburg has a vertically integrated curriculum in cooperation with the University of Groningen in the Netherlands including repetitive clerkships in the ambulatory setting as of the first training year, and the Technical University of Munich (TUM) has a traditional medical curriculum. In total, 952 physicians (Hamburg: 475, Oldenburg: 204, Munich: 269) from all surgical and internal medicine departments present at the three institutions were approached. An invitation with a link to a questionnaire was sent to the physicians by email. The Ethics Committee of the Chamber of Physicians, Hamburg, confirmed the innocuousness of the study and its accordance with the Declaration of Helsinki. Participation was voluntary and anonymized and participants consented to their participation.

We developed an online questionnaire for the ranking of competencies needed for entrustment decisions in first year residents. We collected sociodemographic data, i.e. physicians’ age, gender, position (resident, consultant, supervising attending, department director), and location (Hamburg, Oldenburg, Munich). The residents were not all graduates from the respective medical school. Consultants were board certified specialists with no duties of supervision. Department directors are the heads of the departments who can be substituted by the supervising attendings. Physicians from all four positions are involved clinical work and in teaching activities (lectures, seminars, bedside teaching) in their respective medical school. Their teaching responsibilities are between two and 4 hours a week.

The 25 competencies needed for entrustment decisions in first year residents, which had been defined in an earlier study [16] were placed in random order and their complete definitions [16] were given in an additional window. Instructions for the ranking of the competencies included to drag and drop every competency from the left to the right side of the page and to place them according to their importance for first year residency. Five competencies could be placed in each of the following categories: most important (5 points), very important (4 points), important (3 points), less important (2 points), least important (1 point). There was no ranking within the five categories. When the ranking was completed, completion of the questionnaire had to be confirmed electronically. Between July and September 2016, the survey was open online for 6 weeks in every location. After two and 4 weeks, a reminder was sent by email. For statistical analysis, the median (*Mdn*) was calculated for each competency for different groups (department directors and supervising attendings were merged in one group named “department directors”). When the same median was reached for a competency, the individual rank was given by the underlying mean (*M*). Due to different sample size within the subject-groups, non-parametric tests (Mann-Whitney U test and Kruskal-Wallis test) were used to analyze differences.

## Results

Of the 952 physicians who had been contacted to participate in this study, 286 (30.0%) started and 202 (21.2%) completed the questionnaire. 76 physicians from Hamburg (response rate 15.9%), 44 from Oldenburg (response rate 21.6%), and 82 physicians from Munich (response rate 21.2%) completed the questionnaire. Sociodemographic data of the 202 participants including gender, specialty, and position are shown in Table 1. In total, 73 department directors (age: 47.1 ± 8.4 years), 34 consultants (age: 39.2 ± 5.6 years), and 95 residents (age: 31.5 ± 3.6 years) participated.

**Table 1:** Gender, specialty, and position of all participating physicians (*N* = 202) from Hamburg, Oldenburg, and Munich

	Hamburg (*n* = 76)		Oldenburg (*n* = 44)		Munich (*n* = 82)	
	n	%	n	%	%	n
*Gender*						
Male	45	59.2	38	86.4	60	73.2
Female	29	38.2	6	13.6	22	26.8
Not specified	2	2.6				
*Specialty*						
Surgeons	20	26.3	29	65.9	51	62.2
Internists	56	73.7	15	34.1	31	37.8
*Position*						
Department directors	24	31.7	25	56.8	24	29.3
Consultants	16	21.0	11	25.0	7	8.5
Residents	36	47.3	8	18.2	51	62.2

Table 2 shows the ranking of the competencies relevant for entrustment decisions in first year residents comparing the votes of all physicians according to their university association. No significant differences were found regarding the top 10 competencies between the participating physicians from Hamburg, Oldenburg, and Munich. When all physicians were grouped according to their position (Table 3), consultants were found to rank ‘Active listening to patients’ (*p* < .05) and ‘Written (and digital) account/report to colleagues and supervisors’ (*p* < .01) significantly higher than department directors and residents, while residents ranked ‘Coping with uncertainty’ significantly (*p* < .05) higher than consultants and department directors.

**Table 2:** Ranking order of the 25 competencies by all participating physicians

Competency	Total (N = 202)	Hamburg (n = 76)		Oldenburg (n = 44)		Munich (n = 82)	
	rank	rank	Mdn	rank	Mdn	rank	Mdn
Responsibility	**1**	**1**	5	**1**	5	**2**	5
Knowing and maintaining own personal bounds and possibilities	**2**	**4**	5	**2**	5	**5**	4
Teamwork and collegiality	**3**	**2**	4	**3**	4	**1**	5
Empathy and openness	**4**	**5**	4	**4**	4	**3**	4
Structure, work planning and priorities	**5**	**3**	5	**5**	4	**9**	4
Coping with mistakes	**6**	**6**	4	**7**	4	**8**	4
Active listening to patients	**7**	**8**	3.5	**6**	4	**4**	4
Scientifically and empirically grounded method of working	**8**	**7**	4	**10**	4	**6**	4
Ethical awareness	**9**	**10**	3	**8**	3.5	**7**	4
Verbal communication with colleagues and supervisors	**10**	**9**	3	12	3	**10**	3
Advising patients	11	13	3	**9**	3	12	3
Safety and risk management	12	14	3	11	3	18	3
Active professional development	13	11	3	16	3	16	3
Coping with uncertainty	14	12	3	17	3	17	3
Handling emotions of patients and their relatives	15	18	2.5	13	3	13	3
Adapted informing of patients	16	17	3	15	3	11	3
Respecting privacy and autonomy of the patient	17	19	2.5	18	3	14	3
Attention to individual patient background	18	15	3	14	3	15	3
Written (and digital) account/report to colleagues and supervisors	19	16	3^a^	21	2	19	2
Attention to relatives and caregivers	20	21	2	19	2.5	21	2
Continuity in the care process	21	20	2	20	2	22	2
Attention to psychosocial aspects of health problems	22	23	2	22	2	20	2
Role differentiation	23	22	2	23	1	23	1
Active health promotion	24	24	1	24	1	24	1
Financial and social awareness	25	25	1	25	1	25	1

^a^ *p* < .05; ranks 1 – 10 are marked in bold

**Table 3:** Ranking order of the 25 competencies by all participating physicians according to their position

Competency	Total (N = 202)	Department directors (*n* = 73)		Consultants (*n* = 34)		Residents (*n* = 95)	
	rank	rank	Mdn	rank	Mdn	rank	Mdn
Responsibility	**1**	**1**	5	**1**	5	**2**	5
Knowing and maintaining own personal bounds and possibilities	**2**	**2**	5	**3**	4.5	**5**	4
Teamwork and collegiality	**3**	**3**	4	**4**	4	**1**	5
Empathy and openness	**4**	**4**	4	**2**	5	**4**	4
Structure, work planning and priorities	**5**	**5**	4	**5**	5	**3**	5
Coping with mistakes	**6**	**8**	4	**9**	3	**6**	4
Active listening to patients	**7**	**9**	4	**6**	4^a^	**8**	3
Scientifically and empirically grounded method of working	**8**	**6**	4	11	3	**7**	4
Ethical awareness	**9**	**7**	4	**8**	3	**10**	3
Verbal communication with colleagues and supervisors	**10**	13	3	**10**	3	**9**	3
Advising patients	11	**10**	3	**7**	3.5	13	3
Safety and risk management	12	12	3	14	3	14	3
Active professional development	13	11	3	18	2	12	3
Coping with uncertainty	14	18	2	17	3	11	3*
Handling emotions of patients and their relatives	15	16	3	12	3	15	3
Adapted informing of patients	16	17	3	15	3	16	3
Respecting privacy and autonomy of the patient	17	15	3	16	3	18	2
Attention to individual patient background	18	14	3	19	2	17	3
Written (and digital) account/report to colleagues and supervisors	19	20	2	13	3^b^	19	2
Attention to relatives and caregivers	20	19	2	21	2	20	2
Continuity in the care process	21	21	2	20	2	22	2
Attention to psychosocial aspects of health problems	22	23	2	22	2	23	2
Role differentiation	23	22	1	24	1	21	2
Active health promotion	24	24	1	23	1	24	2
Financial and social awareness	25	25	1	25	1	25	1

^a^ *p* < .05, ^b^ *p* < .01; ranks 1 – 10 are marked in bold

Grouped according to specialty, among the top 10 competencies, internists attributed significantly higher importance to ‘Structure, work planning and priorities’ significantly (*p* < .05) than surgeons while surgeons evaluated ‘Verbal communication with colleagues and supervisors’ significantly (*p* < .05) higher than internists (Table 4). Furthermore, internists ranked ‘Attention to psychosocial aspects of health problems’ significantly (*p* < .05) higher compared with surgeons. Within the top 10 competencies, women evaluated ‘Verbal communication with colleagues and supervisors’ and ‘Structure, work planning and priorities’ significantly (*p* < .01) higher than men (Table 5). In contrast, men ranked ‘Scientifically and empirically grounded method of working’ significantly (*p* < .01) than women.

**Table 4:** Ranking order of the 25 competencies by all participating surgeons and internists

Competency	Surgeons (*n* = 100)		Internists (*n* = 102)	
	rank	Mdn	rank	Mdn
Responsibility	**1**	5	**1**	5
Teamwork and collegiality	**2**	5	**3**	4
Knowing and maintaining own personal bounds and possibilities	**3**	5	**5**	4
Empathy and openness	**4**	4	**4**	4
Structure, work planning and priorities	**5**	4	**2**	5^a^
Coping with mistakes	**6**	4	**7**	4
Active listening to patients	**7**	4	**8**	4
Scientifically and empirically grounded method of working	**8**	4	**6**	4
Verbal communication with colleagues and supervisors	**9**	4^a^	**10**	3
Ethical awareness	**10**	4	**9**	4
Advising patients	11	3	13	3
Safety and risk management	12	3	14	3
Active professional development	13	3	11	3
Coping with uncertainty	14	3	12	3
Handling emotions of patients and their relatives	15	3	15	3
Adapted informing of patients	16	3	18	3
Respecting privacy and autonomy of the patient	17	3	16	3
Attention to individual patient background	18	3	17	3
Attention to relatives and caregivers	19	2	20	2
Written (and digital) account/report to colleagues and supervisors	20	2	19	2
Continuity in the care process	21	2	22	2
Attention to psychosocial aspects of health problems	22	2^a^	23	2
Role differentiation	23	1	21	2
Active health promotion	24	1	24	1
Financial and social awareness	25	1	25	1

^a^ *p* < .05; ranks 1 – 10 are marked in bold

**Table 5:** Ranking order of the 25 competencies by all participating women and men

Competency	Women (*n* = 57)		Men (*n* = 143)	
	rank	Mdn	rank	Mdn
Responsibility	**1**	5	**1**	5
Structure, work planning and priorities	**2**	5^b^	**5**	4
Knowing and maintaining own personal bounds and possibilities	**3**	5	**3**	4
Teamwork and collegiality	**4**	4	**2**	4
Empathy and openness	**5**	4	**4**	4
Verbal communication with colleagues and supervisors	**6**	4^b^	13	3
Active listening to patients	**7**	4	**9**	4
Coping with mistakes	**8**	4	**7**	4
Ethical awareness	**9**	3	**8**	4
Coping with uncertainty	**10**	3	16	3
Scientifically and empirically grounded method of working	11	3	6	4^b^
Advising patients	12	3	**10**	3
Safety and risk management	13	3	11	3
Handling emotions of patients and their relatives	14	3	14	3
Active professional development	15	3	12	3
Adapted informing of patients	16	3	18	3
Attention to individual patient background	17	3	17	3
Role differentiation	18	3	22	1
Respecting privacy and autonomy of the patient	19	2	15	3
Written (and digital) account/report to colleagues and supervisors	20	2	20	2
Attention to relatives and caregivers	21	2	19	2^a^
Continuity in the care process	22	2	21	2
Attention to psychosocial aspects of health problems	23	2	23	2
Active health promotion	24	1	24	1
Financial and social awareness	25	1	25	1

^a^ *p* < .05, ^b^ *p* < .01; ranks 1 – 10 are marked in bold

## Discussion

The top 10 competencies for entrustment decisions in first year residents were not significantly different between physicians from three medical schools with different undergraduate curricula. Our finding is in line with the data from a previous ranking study conducted with clinical educators from two different countries and medical schools with different curricula, which also showed no significant differences among the top 10 competencies [15]. We found 80% congruency of the 10 key competencies identified in our study with the 10 key competencies found in the ranking study performed with physician educators from two European countries [15] and 90% congruency with the top 10 competencies of the German cohort from this study [15]. This high level of agreement within a large cohort of physicians from different medical schools on competencies needed by first year residents might be a first step towards a standardization of competency-based medical education and the resulting demand for faculty development with respect to teaching and assessing of competencies [17]. Aspects of management, communication, and professionalism were identified among the top 10 competencies, representing each CanMEDS role except the role of ‘Health advocate’ [3].

On the other hand, we found significant differences between consultants, department directors and residents regarding the ranking of three competencies. Consultants assessed ‘Active listening to patients’ and ‘Written (and digital) account/report to colleagues and supervisors’ significantly more relevant for first year residents than department directors and the residents themselves. In their interaction with residents on the ward, consultants might be more aware than department directors and residents that history-taking alone, which requires active listening to a patient, can lead to the correct diagnosis in more than 80% [18]. The importance of effective history-taking has also been recognized as a valued competency for quality patient care in internal medicine residency programs [19]. From their daily practice, consultants seem to be much more aware of the importance of a resident’s competency to write reports, as the quality of discharge summaries is often inadequate concerning timeliness, transmission, and content [20]. A meta-analysis of the literature revealed many options to improve the competency of written communication in residency training [21].

Residents ranked ‘Coping with uncertainty’ significantly higher than consultants and department directors. This competency is not easy to acquire during undergraduate medical training, but residents might observe its importance in daily practice during their training. Patients’ recollections of their symptoms and history, for instance, can be inconsistent when inquired by different physicians [22], which can cause feelings of uncertainty in first year residents who have not encountered this difference during their time at medical school. Consultants might not be aware of this difference to cause anxiety anymore because they have become used to coping with uncertainty in their daily work. Furthermore, residents working on a ward become aware of the necessity to cope with uncertainty in clinical decision making, which will persist even with increasing medical knowledge [23]. Heuristics might help residents to become more confident with their clinical decisions [24] and at the same time convince them that uncertainty may persist and that it is important to develop the competency to tolerate it during the advancing in postgraduate training.

Among the top 10 competencies, internists ranked ‘Structure, work planning and priorities’ significantly higher than surgeons. For internists, close interprofessional interaction with nursing staff has been found to involve especially patient management and clinical reasoning competencies [25], which are important qualities to run a ward successfully. In contrast, surgeons ranked aspects of communication significantly higher than internists, which could be related to the great impact communication failures may have on patient safety in the context of surgery [26]. One relevant example for surgical communication supporting patient outcome are team time-outs with checklists implemented in the operating theatre [27]. These differences in competencies relevant for entrustment decisions between internist and surgeons could be relevant when selecting physicians for different residency programs. In our study, female physicians rank competencies regarding management and communication in first year residents significantly higher than male physicians while male physicians rank ‘Scientifically and empirically grounded method of working’ higher than female physicians. These differences in the assessment of residents’ competencies by male and female physicians could imply that supervision of residents might differ between male and female supervisors and it underscores the findings of differences in patient care by male and female physicians [28]. Furthermore, in a study among junior faculty, physicians on role models for physician-scientists, women attributed more importance to communication and management abilities in their role models, whereas male physicians envisioned scientific knowledge as important in their role models [29]. These gender differences in the ranking of competencies for first year residents might be relevant for more gender specific training during residency, e.g. more communication training for male residents and more training in scientific work for female residents.

A strength of our study is the large number of experienced physicians who participated in the ranking of competencies for the first year of residency. However, the response rate of 21.2% was only moderate. Furthermore, since only surgeons and internists were included in the study, the generalizability to physicians of other specialties is limited. In addition, women and consultants were underrepresented in our study. Additionally, the number of physicians from the three different medial faculties was different. Despite these limitations, the results of our study are consistent with an earlier study with few participants [15] and provide additional information regarding the importance of certain competencies relevant for entrustment decisions in first year residents.

## Conclusions

Physicians from medical faculties with different undergraduate curricula show no differences in the ranking of competencies relevant for entrustment decisions in first year residents. Their focus lies on competencies regarding professional behavior, communication, and patient management. Especially aspects of medical professionalism prevail among the top 10 competencies, like showing responsibility for the work. Differences occur between the perspectives of department directors, consultants, and residents as well as between surgeons and internists and male and female physicians. These differences should be considered carefully in the planning of competence-based postgraduate education programs.
